# Decolorization of industrial synthetic dyes using engineered *Pseudomonas putida* cells with surface-immobilized bacterial laccase

**DOI:** 10.1186/1475-2859-11-75

**Published:** 2012-06-11

**Authors:** Wei Wang, Zhen Zhang, Hong Ni, Xiaomeng Yang, Qianqian Li, Lin Li

**Affiliations:** 1 State Key Laboratory of Agricultural Microbiology, Huazhong Agricultural University, Hubei, Wuhan, 430070, China; 2 School of Life Sciences, Hubei University, Hubei, Wuhan, 430062, China; 3 Key Laboratory of Subtropical Agricultural Resource and Environment, Ministry of Agriculture, Huazhong Agricultural University, Wuhan, 430070, China

**Keywords:** Dye decolorization, Laccase, Cell surface display, *Pseudomonas putida*, Whole-cell biocatalyst

## Abstract

****Background**:**

Microbial laccases are highly useful in textile effluent dye biodegradation. However, the bioavailability of cellularly expressed or purified laccases in continuous operations is usually limited by mass transfer impediment or enzyme regeneration difficulty. Therefore, this study develops a regenerable bacterial surface-displaying system for industrial synthetic dye decolorization, and evaluates its effects on independent and continuous operations.

****Results**:**

A bacterial laccase (WlacD) was engineered onto the cell surface of the solvent-tolerant bacterium *Pseudomonas putida* to construct a whole-cell biocatalyst. Ice nucleation protein (InaQ) anchor was employed, and the ability of 1 to 3 tandemly aligned N-terminal repeats to direct WlacD display were compared. Immobilized WlacD was determined to be surface-displayed in functional form using Western blot analysis, immunofluorescence microscopy, flow cytometry, and whole-cell enzymatic activity assay. Engineered *P. putida* cells were then applied to decolorize the anthraquinone dye Acid Green (AG) 25 and diazo-dye Acid Red (AR) 18. The results showed that decolorization of both dyes is Cu^2+^- and mediator-independent, with an optimum temperature of 35°C and pH of 3.0, and can be stably performed across a temperature range of 15°C to 45°C. A high activity toward AG25 (1 g/l) with relative decolorization values of 91.2% (3 h) and 97.1% (18 h), as well as high activity to AR18 (1 g/l) by 80.5% (3 h) and 89.0% (18 h), was recorded. The engineered system exhibited a comparably high activity compared with those of separate dyes in a continuous three-round shake-flask decolorization of AG25/AR18 mixed dye (each 1 g/l). No significant decline in decolorization efficacy was noted during first two-rounds but reaction equilibriums were elongated, and the residual laccase activity eventually decreased to low levels. However, the decolorizing capacity of the system was easily retrieved via a subsequent 4-h cell culturing.

****Conclusions**:**

This study demonstrates, for the first time, the methodology by which the engineered *P. putida* with surface-immobilized laccase was successfully used as regenerable biocatalyst for biodegrading synthetic dyes, thereby opening new perspectives in the use of biocatalysis in industrial dye biotreatment.

## **Background**

Laccases (EC 1.10.3.2, benzenediol:oxygen oxidoreductase) are a diverse family of multi-copper enzymes that oxidize a broad range of aromatic compounds including orthodiphenols, *p*-dihydroxybenzenes, aminophenols, polycyclic aromatic hydrocarbons, and aromatic polyamines [1]. They are also involved in diverse biochemical processes, such as oxidization of various non-aromatic compounds and metal oxides, plant lignification, cellular pigment production, fungal pathogenesis, and resistance to UV and H_2_O_2_, among others [2-4]. These enzymes have received particular interest in commercial applications of treating industrial phenolic substrates because they are biodegradable, cost-effective, and environmentally friendly [5-7].

Laccases are widely found in fungi, plants, and various bacteria [8,9]. Although fungal and bacterial laccases have similar structures, their amino acid sequences are quite different [10]. In addition, bacterial laccases often occur as monomers, whereas certain fungal laccases occur as isoenzymes that normally oligomerize to form multimeric complexes [10,11]. In recent years, bacterial laccases have gained increasing attention for their potential in biodegrading environmentally important phenolic pollutants because of their relatively high production rate, high thermostability, and wider pH range, among others [12,13].

Textile dyes are a class of highly diverse chemicals, which includes numerous organic chromophoric compounds and synthetic products, typically the azo-, anthraquinone-, indophenol-, triphenylmethane-, and heterocycle-containing dyes, among others [6,14]. Contamination caused by textile dyes from industrial effluent has become a major environmental concern, because these dyes are toxic or are cross-coupled into toxic or carcinogenic metabolites and are relatively recalcitrant to degradation, significantly threatening the ecosystem [6,15]. Conventional treatments for textile effluent that include physicochemical methods, such as the use of activated carbon, chemical flocculation, and filtration or coagulation [16], have been proven uneconomical or ineffective [7]. Alternatively, biological process based on laccases is a promising solution for treating such effluents. Several previous investigations using fungal laccases have demonstrated the feasibility of such approaches [6,7]. However, fungal laccases have disadvantages, especially low production rate and complicated enzyme regenerability although they are predominant in the biotreatment of textile dyes [17]. The use of bacterial laccases has recently opened new perspectives on these applications. Several studies have described bacterial laccase-based approaches, such as *Bacillus* spore-bound laccase used at high temperatures and pH values [12], a laccase from *Streptomyces coelicolor* used under alkaline conditions [5], mutated *Bacillus licheniformis* CotA variants with high expression level and high activity [18], and laccase-active bacterial consortiums used for bioremediation of various textile dyes [16,19-21].

A successful system for laccase-based textile dye biodegradation should ensure enzyme functionality and maximize catalytic efficiency in high pollutant concentrations. Moreover, the facile regeneration capacity for continuous application of the enzyme would be particularly valuable for such a system. Although certain previous strategies using immobilized or spore-bound bacterial laccases have shown enzymatic decolorization effects [12,13], restriction for the reuse of matrix-immobilized enzymes and possible shortcomings for spore-bound laccases, such as substrate slack or limited diffusion caused by their exosporium barriers should also be considered. By contrast, our previous work showed that bacterial cell surface-immobilized laccase was efficient in oxidizing phenolic substrates and is, especially, a regenerable whole-cell catalyst [22]. Hence, the bacterial surface display of laccase has been considered a better alternative for degrading toxic dyes from textile effluents.

The surface display of foreign enzyme proteins on live bacterial cells allows direct enzymatic reaction on cell surface, eliminating mass transfer limitation and increasing reaction rates [23,24]. Moreover, a stable and regenerable cell platform is apparently conducive to retain the activity of surface-displayed enzymes [25,26]. Bacterial display systems are normally grouped into those that allow N-terminal, C-terminal, and “sandwich” fusions. These fusions are achieved by genetically incorporating heterologous protein with various anchoring proteins that have transmembrane transport activity and capacity to bind to outer membranes as well as those surface-appendiculate structures. Among these anchoring proteins, ice nucleation protein (INP) from *Pseudomonas syringae* has been generally regarded as one of the most efficient anchor proteins for Gram-negative bacteria [24,27]. Previous studies have shown that both full-length and truncated INP variants can immobilize target proteins [28,29]. Therefore, INP-anchored system has been mostly used to display peptides or proteins of various Gram-negative bacteria because of the broad availability of this anchor. However, the INP-mediated surface display method has not been used thus far to improve the catalytic efficiency of bacterial laccases although various reports have described the successful application of INP-anchored functional proteins.

Synthetic dyes represent the largest class of dyes applied in the textile and dyeing industries [30]. These dyes cannot be easily removed from effluents via conventional sewage treatment or readily degraded under natural conditions. In this study, the N-terminal moiety of a newly identified INP (InaQ) was used as the anchoring motif to display the fusion protein with a mutated bacterial laccase (WlacD) onto the surface of solvent-tolerant *P. putida* AB92019 cells. The expression, as well as surface localization, of fusion proteins with 1 to 3 tandemly aligned InaQ-N repeats and WlacD in the engineered *P. putida* cells were analyzed using several assays. The enzymatic activity of intact cells expressing these fusion enzymes was comparatively determined. The optimized engineered strain was then applied to decolorize two synthetic dyes. The relative decolorization levels of these dyes, either in separate or combined form, and the decolorizing effect, as well as regenerability of the system, in a continuous three-round shake-flask trial was investigated.

## **Results**

### **Construction and expression of InaQ-N/WlacD fusion proteins**

**Figure 1:**
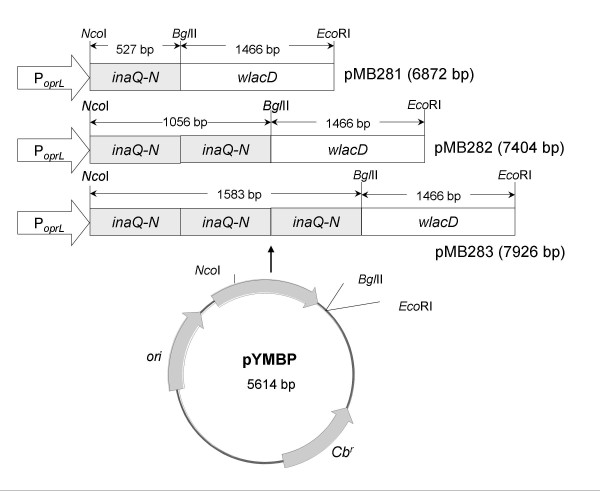
**Map of recombinant plasmids.** Plasmid pYMBP was used as the parent vector to construct pMB281, pMB282, and pMB283. *P*_*oprL*_, a constitutive promoter in *P. putida*; *Cb*^*r*^, carbenicillin-resistant gene; *ori*, replication origin of *Pseudomonas* sp.; *inaQ-N*, N-terminal domain of *inaQ*; and *wlacD*, a mutated bacterial laccase gene.

Transformed *P. putida* strains harboring recombinant plasmids pMB281, pMB282, and pMB283 that encode InaQ-N/WlacD, (InaQ-N)_2_/WlacD, and (InaQ-N)_3_/WlacD, respectively, were constructed to express these fusion proteins during the growth phase. The binary and tripartite tandemly aligned *inaQ-N* repeats were fused with the bacterial laccase gene *wlacD*[31] to create fusion genes at the whole encoding frame controlled under a constitutively active promoter *P*_*oprL*_ (Figure 1).

**Figure 2:**
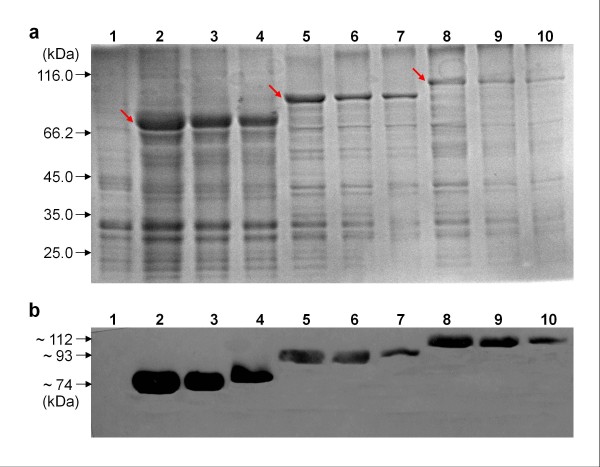
**SDS-PAGE analysis of recombinant*P. putida*strains (a) and Western blot analysis of recombinant*P. putida*strain cell fractions (b).** Panels (**a**) and (**b**): lane 1, *P. putida* AB92019 (negative control); lanes 2–4, whole cell fraction (WC), cytoplasmic fraction (CP), and outer membrane fraction (OM) of *P. putida* MB284 expressing InaQ-N/WlacD, respectively; lanes 5–7, WC, CP, and OM of *P. putida* MB285 expressing (InaQ-N)_2_/WlacD, respectively; lanes 8–10, WC, CP, and OM of *P. putida* MB286 expressing (InaQ-N)_3_/WlacD, respectively.

Expression patterns of fusion proteins in transformed *P. putida* MB284, MB285, and MB286 cells were detected by sodium dodecyl sulfate polyacrylamide gel electrophoresis (SDS-PAGE). The profile showed that fusion proteins, InaQ-N/WlacD, (InaQ-N)_2_/WlacD, and (InaQ-N)_3_/WlacD, were synthesized accurately with predicted molecular masses of ~ 74 (Figure 2a, lane 2, indicated by arrow), ~ 93 (Figure 2a, lane 5, indicated by arrow), and ~ 112 kDa (Figure 2a, lane 8, indicated by arrow), respectively. Equal volumes of subcellular fractions from the very late growth phase of each *P. putida* construct (24 h) were prepared and then used for SDS-PAGE analysis. Considerable amounts of fusion proteins were also retained intracellularly (Figure 2a, lanes 3, 6, and 9) in addition to outer membrane fraction (OM) complex-targeted fusion proteins in the OM fractions of the three constructs (Figure 2a, lanes 4, 7, and 10).

The Western blot profile of the expressed proteins revealed clear signs of all corresponding proteins from subcellular fractions of *P. putida* MB284 (~74 kDa, Figure 2b, lanes 2 to 4), MB285 (~93 kDa, Figure 2b, lanes 5 to 7), and MB286 (~112 kDa, Figure 2b, lanes 8 to 10), whereas none was found in the control (Figure 2b, lane 1).

### **Surface localization analysis of fusion proteins**

**Figure 3:**
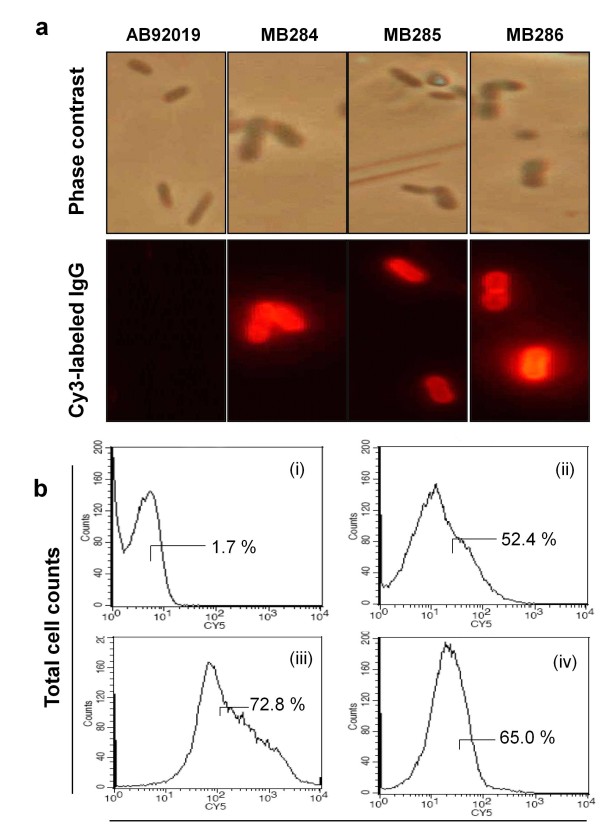
**Micrographs (a) and flow cytometric analysis (b) of recombinant*P. putida*strains.** The cells were treated with anti-WlacD polyclonal antiserum followed by secondary Cy3-conjugated goat anti-mouse IgG for immunofluorescence microscopic examination or with goat anti-mouse Cy5-conjugate antibody for flow cytometric analysis. Panel (b): (i) *P. putida* AB92019 (negative control); (ii) *P. putida* MB284; (iii) *P. putida* MB285; and (iv) *P. putida* MB286. A total of 100,000 cells were analyzed for each flow cytometry experiment.

The surface localization of fusion proteins InaQ-N/WlacD, (InaQ-N)_2_/WlacD, and (InaQ-N)_3_/WlacD expressed in transformed *P. putida* MB284, MB285, and MB286, respectively, was analyzed using Western blot analysis, immunofluorescence microscopy, and flow cytometry. All the OM-complex fractions of the three constructs exhibited protein bands corresponding to those present in whole cell fraction (WC) and cytoplasmic fraction (CP) samples (Figure 2a, lanes 4, 7, and 10). This result indicates the surface localization of fusion proteins in the transformed cells. The Cy3 fluorescence on the cell surface of MB284, MB285, and MB286 is illustrated by Figure 3a, verifying the surface localization of proteins caused by the inability of Cy3-labeled antibodies to penetrate the outer membrane of the cells. This phenomenon is consistent with the results shown by the FACS assay, in which pronounced Cy5 fluorescence intensity of the intact transformed cells expressing fusion proteins [Figure 3b(ii), (iii), and (iv)] was confirmed. The results in Figure 2 and 3 indicate that fusion proteins were immobilized successfully onto the surface of target cells.

### **Effect of different tandem-aligned anchors on display efficiency**

The transformed *P. putida* cells expressing fusion proteins comprised single, two, or three tandem-aligned InaQ-N repeats, and laccase WlacD were compared through their display efficiencies using flow cytometry analysis. An increase in the anchoring motifs in the two or three InaQ-N repeats enhanced surface immobilization efficiency over that of single InaQ-N fusion protein [72.8% and 65.0% vs. 52.4%, respectively; Figure 3b(iii), (iv), and (ii)]. However, increasing the InaQ-N repeats to three did not result in a corresponding increase in surface immobilization efficiency compared with that of the two InaQ-N repeats, as indicated by the greater surface-immobilization efficiency of the anchoring motif with two tandem aligned repeats [Figure 3b(iii)] compared with the (InaQ-N)_3_ anchor [Figure 3b(iv)].

### **Whole-cell laccase activity of recombinant strains**

**Figure 4:**
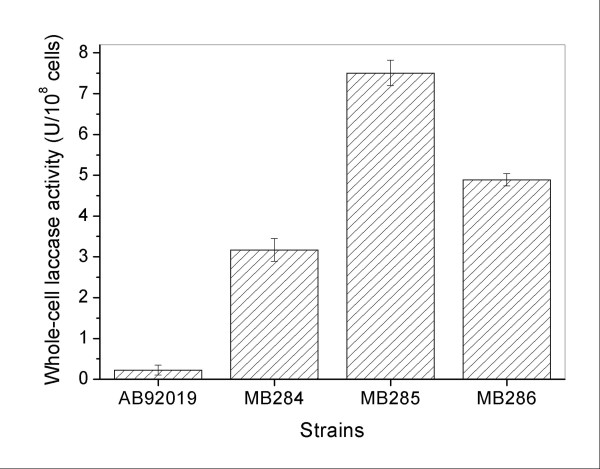
**Measurement of whole-cell laccase activity of recombinant*P. putida*strains.** The recipient strain, *P. putida* AB92019, was used as the negative control. Each value and error bar represents the mean and standard deviation of three independent experiments.

Whole-cell laccase activity of recombinant strains was determined using ABTS as the substrate to verify whether surface-immobilized fusion enzymes conferred laccase activity. All three recombinant strains exhibited increased enzymatic activity compared with the control strain, indicating that this oxidase substantially retained its activity even under N-terminus-fused and surface-targeted confirmation (Figure 4). The enzymatic activity of the three recombinant strains was significantly modulated by the amount of surface-displayed fusion proteins, as observed in MB285 cells with the highest whole-cell enzyme activity that resulted from its highest immobilization efficiency, as confirmed by FACS assay (Figure 3b).

### **Decolorization of separate anthraquinone-dye and azo-dye by*P. putida*MB285 cells**

**Figure 5:**
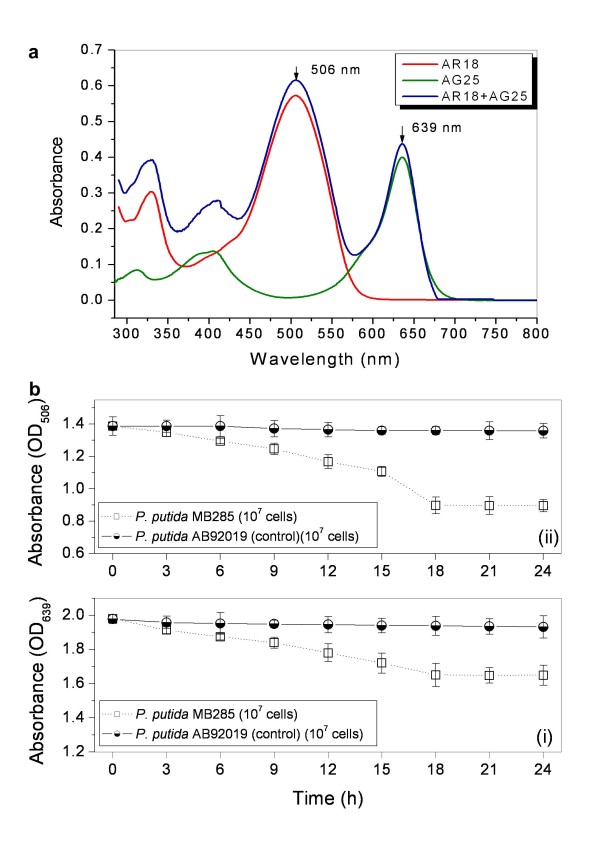
**Dye decolorization of*P. putida*MB285 cells towards independent AG25 and AR18.** (**a**) Full wavelength (290 nm to 800 nm) scanning curves of AG25 and AR18 indicate the maximal adsorbent peaks, which were used as OD values for dye absorbance measurement. (**b**) A final 10^7^ cells in each reaction mixture was measured. (i) Effect on AG25; (ii) Effect on AR18.

Two industrially used synthetic acid dyes, AG25 and AR18, respectively representing anthraquinone- and azo-dyes, were selected as decolorization substrates to evaluate the dye decolorization efficacy of the recombinant *P. putida* MB285. Prior to the measurement, the full wavelength absorption spectra (from 290 nm to 780 nm) of the dyes (separate or combined) were recorded, given the maximum spectral peaks at 639 nm for AG25 and 506 nm for AR18 (Figure 5a). No new peak occurred for the mixed AG25/ AR18 dye. A relatively low MB285 cell concentration (approximately 1.5 × 10^7^ cells in the reaction mixture) was tested for the decolorization level for the dye decolorization experiments. Under given conditions, the MB285 cells required 18 h to reach maximum decolorization of AG25 [Figure 5b(i)], which can be expressed as the relative decolorization value of 35.5%. Interestingly, MB285 cells exhibited the decolorization effect on this dye without any mediator although the azo-like dye AR18 is not the common substrate for laccase catalytic reaction [Figure 5b(ii)]. Relative decolorization value was recorded as 17.0%. By contrast, control cells had limited relative decolorization value of less than 2% for both AG25 and AR18 with similar time courses.

### **Dye decolorization of separate AG25 and AR18 by*P. putida*MB285 cells without Cu**^**2+**^**and different temperature and pH conditions**

**Figure 6:**
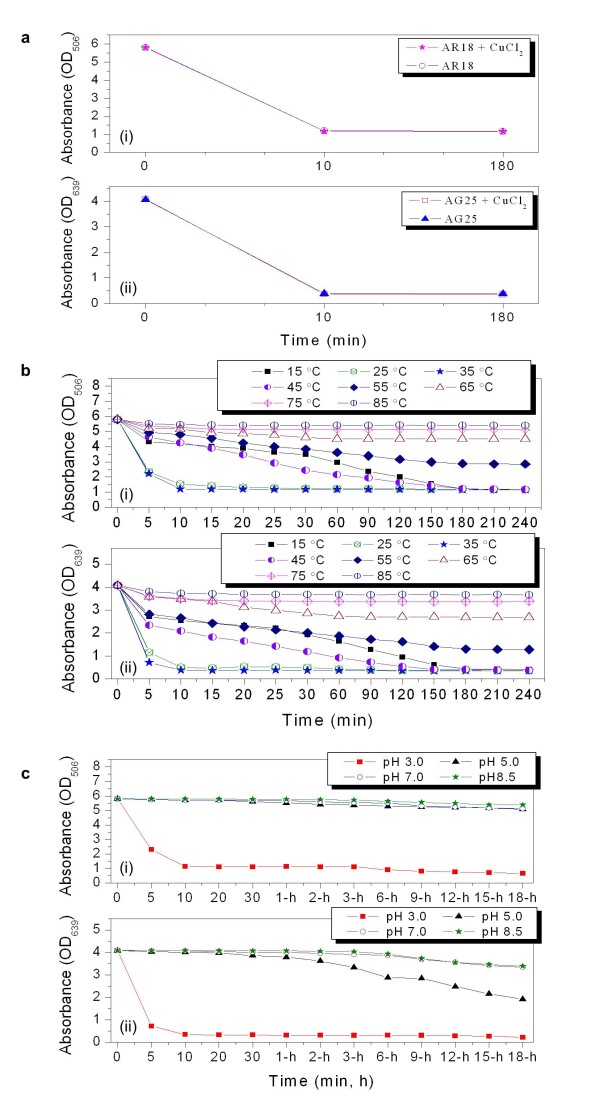
**Effect of Cu**^**2+**^**(a), temperature (b), and pH value (c) on AG25 and AR18 decolorization.** A final concentration of 1 × 10^9^ cells and 1 g/l of dye substrate in each reaction mixture were measured. (i) Effect on AR18; (ii) Effect on AG25.

To investigate whether Cu^2+^ is required for decolorization by the engineered *P. putida* cells, the decolorization of separate AG25 and AR18, each at industrially applied concentration of 1 g/l, was examined with or without adding Cu^2+^ into the reaction mixtures. Under the given conditions (approximately 1 × 10^9^ cells/ml, pH 3.0, 25°C, and 0.1 mmol/l Cu^2+^ for treated samples), the MB285 cells exhibited similar decolorization patterns of AR18 [Figure 6a(i)] or AG25 [Figure 6a(ii)] with and without Cu^2+^, indicating that the decolorization of both dyes was Cu^2+^-independent.

The influence of temperature on decolorizing efficacy was evaluated across the range of 15°C to 85°C. The results showed that decolorization of both AR18 [Figure 6b(i)] and AG25 [Figure 6b(ii)] was conducted at 35°C and 25°C with relatively high reactivity than those at other temperatures, in which a relative decolorization of 73.8% and 61.7% for AR18, 87.5% and 82.4% for AG25 were reached after only 5 min. Each highest relative decolorization value was recorded at 35°C in the experimental time-course. A relatively steady decolorization of AR18 and AG25 was also observed at 15 and 45°C, where almost equivalent decolorization values with those at 35 and 25°C were achieved in 3 h. However, decolorization appeared to be ineffective at temperatures higher than 65°C.

Figure 6c showed that the decolorization of either AR18 or AG25 required an obligate pH value of 3.0, and the activity was apparently lower for AR18 than that for AG25 based on other pH values.

**Figure 7:**
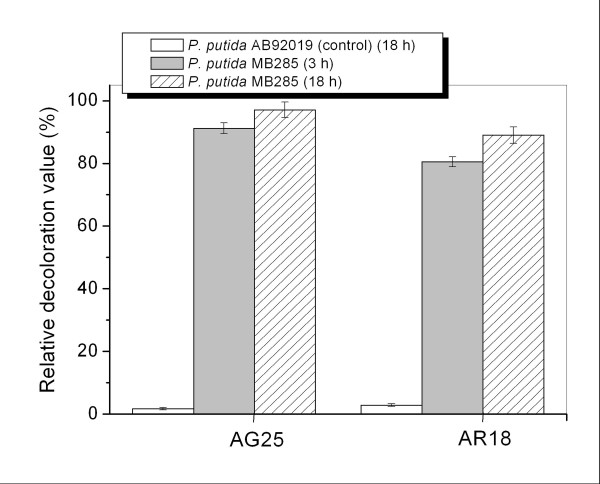
**Decolorization values of AG25 and AR18 with*P. putida*MB285 cells.** Decolorization was catalyzed by 1 × 10^9^ cells (each reaction) for 3 h or 18 h at 35°C and pH 3.0. *P. putida* AB92019 strain was used as control.

Based on these data, reaction was set up under optimized conditions (1 × 10^9^ cells, 1 g/l dye, 35°C, and pH 3.0) to evaluate the relative decolorization levels of AG25 and AR18. As was shown in Figure 7, in addition to a high decolorization value of 91.2% in 3 h and 97.1% in 18 h toward AG25, a high activity to AR18 by 80.5% (3 h) and 89.0% (18 h) without any mediator was recorded.

### **Continuous decolorization of the AG25/AR18 mixed dye**

**Figure 8:**
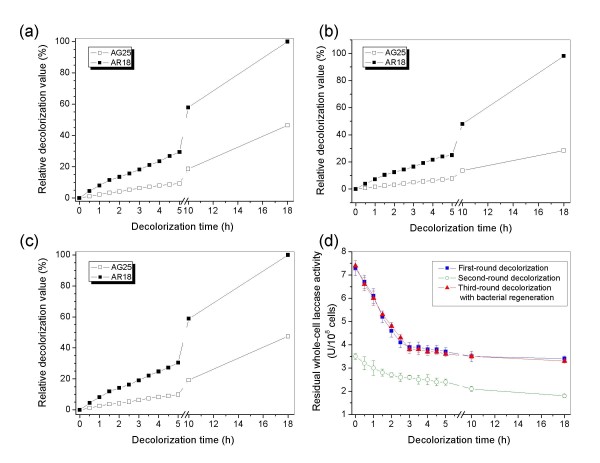
**Continuous decolorization of AG18/AG25 mixed dyes (each 1 g/l at final concentration).** Panels (**a**) and (**b**), continuous first- and second-round decolorization reactions; Panel (**c)**, third-round decolorization after 4-h cultivation of cultures by directly adding LB medium into the flask; Panel (**d**), residual who-cell laccase activity of *P. putida* MB285 cells after each-round decolorization reaction.

Real industrial effluents usually include mixtures of several dyes. Continuous three-round decolorization experiments on the activities of residual laccase after decolorization reaction, as well as the regenerability of engineered cells, were performed in laboratory shake-flask trials. The AG25/AR18 mixed dye was used as substrate (equal aliquot at final concentration of 1 g/l each). Although a longer time was required for the reaction equilibrium in contrast to that of the decolorizing reaction of separate dyes, *P. putida* MB285 cells exhibited high activity to AG25 by 99.9% (18 h) and a substantial activity to AR18 by 46.5% (18 h) (Figure 8).

The whole-cell laccase activity of *P. putida* MB285 cells was monitored during the decolorization time-course, indicating a steadily decreasing pattern at each round of decolorization (Figure 8d). Interestingly, the cells still maintained high decolorization activity to AG25 by 98.0% (in the second 18 h) and an activity to AR18 by 28.5% (in the second 18 h) in the continuous second-round decolorization reaction although the residual whole-cell laccase retained only approximately 50% of the initial activity after the first-round decolorization reaction in 18 h (Figure 8d). However, the residual whole-cell laccase activity was reduced to a low level (approximately 20% of the initial activity of first-round decolorization) after the second-round reaction (in the second 18 h) (Figure 8d), suggesting that engineered cells are incapable of further dye degradation.

The reaction solution was removed and the remaining cells were cultured for 4 h by directly adding LB medium to the residual cell inocula to regenerate the decolorizing activity of the system. The decolorization efficacy of either AG25 (99.9%, 18 h) or AR18 (47.5%, 18 h) was retrieved at the first-round level (Figure 8c) in the third-round decolorization reaction with regenerated bacteria. The data of the residual laccase activity after regenerative culturing and the third-round reaction were in agreement with that of the cells after first-round decolorization reaction (Figure 8d).

## **Discussion**

The display of heterologous proteins on the surface of target bacteria has been accomplished over the past decade, exhibiting promising prospects in several biotechnological processes. In the present study, an *in vitro* genetically modified bacterial laccase WlacD was functionally immobilized onto the surface of *P. putida* cells through an optimized ice nucleation protein anchor. This system was then used as a whole-cell biocatalyst to degrade two industrially used synthetic dyes in laboratory trials. The significant decolorization effect on the tested dyes using the optimized laccase-displaying system, together with their distinctive features, such as regenerability and eliminability of mass transfer limitation or passive diffusion of substrates, suggests the potentials of this strategy in textile dye effluent treatment. To the best of our knowledge, this study is the first approach to decolorize industrial synthetic dyes using engineered bacterial cells with surface-immobilized laccase.

*P. putida* is a well-known solvent-tolerant bacterium capable of utilizing a wide range of inorganic and organic compounds, rendering it an attractive host for developing cell surface display systems for environmental or biotechnological applications. Several previous studies have described the methodology by which surface-immobilized heterologous enzymes or other proteins can be used as whole-cell catalysts [25,28,32-34] or bioadsorbents [35]. In these approaches, the full-length or truncated INP anchors from *P. syringae* were mostly utilized as anchoring motifs to construct various surface display systems. INP-mediated surface display systems have been extensively exploited from *Escherichia coli* to *Pseudomonas* sp., and *Vibrio* sp., among others [23,24,27]. However, insufficient surface-bound target proteins less than 50% of the total intracellularly expressed proteins either in *E. coli*[35,36] or in *P. putida*[34,35] remains to be improved. In the current study, two and three tandem-aligned InaQ-Ns were employed as combined anchors to compare the surface-displaying activity of fusion incorporations to promote surface display efficiency of an INP-mediated system. Successful display of 665 aa (InaQ-N/WlacD), 840 aa [(InaQ-N)_2_/WlacD], and 1015 aa [(InaQ-N)_3_/WlacD] proteins using the increased InaQ-N anchors improved the display efficiency by increasing the number of anchor proteins (Figure 2,3 and 4). However, these results also revealed that the translocation and transport of fusion proteins mediated by InaQ-N can be limited to certain amino acid residues. Thus, transport and surface binding should be coordinated, as was indicated by the highest display efficiency exhibited by the two tandem aligned, in contrast to the lower activity of those with single InaQ-N anchor and the decreased activity when the anchor numbers were increased to three (Figure 3 and 4). Theoretically, numerous anchoring motifs probably contributed to the increase in surface binding efficiency, thus, the exceeding length of the fusion protein (in the case of three InaQ-N repeats) may have also decreased the transmembrane and transport activity while targeting the cell surface.

We have previously engineered *B. thuringiensis* vegetative cells and spores with surface-immobilized similar laccase, which demonstrated that laccase can be targeted onto the cell surface without radically altering enzymatic activity [22,37]. Compared with the *B. thuringiensis* system, the current *P. putida* system demonstrates several advantages. First, it exhibits a relatively high whole-cell enzymatic activity. Although the activity of *B. thuringiensis* vegetative cells seems comparable, a low whole-spore activity has been observed for spores. However, spores are the main growth phase of *B. thuringiensis* in natural environments, and its vegetative cells are typically converted autogenetically into spores under adverse conditions. Second, the *P. putida* system mediated by InaQ-N allows the display of relatively large proteins (over 1,000 residues in length), in contrast to less than 500 residues for the *B. thuringiensis* system [22]. Hence, the developed *P. putida* system is more suitable for dye decolorization.

The transformed *P. putida* MB285 cells exhibited high whole-cell enzymatic activity against anthraquinone dye AG25 and a remarkable activity against diazo dye AR18, suggesting that surface-immobilized laccases were anchored stably in the functional confirmation (Figures 5,6 and 7). These results are in agreement with previously reported purified fungal laccases, which showed extremely high activity in the decolorization of anthraquinone-like dyes [38,39], but reduced activity against azo-like dyes [40]. This finding may be attributed to the different structural features of the dyes, i.e., unlike azo dyes, anthraquinone dyes are direct substrates of laccase-based oxidation. Interestingly, the control strain *P. putida* AB92019 exhibited a slightly higher decolorization activity for AR18 than that for AG25 (Figure 7). Several previous investigations have reported that certain *Pseudomonas* strains produced azo-dye-degrading enzymes, such as azoreductase [41,42]. Therefore, it is of interest to identify whether the background degrading activity of AR18 reflects a low level expression of azoreductase in the control strain.

In natural environments, certain synthetic dyes, such as azo dyes, are particularly recalcitrant to decolorization. Although laccase-based oxidation can be utilized to provide new approaches, previous investigations revealed that effective degradation of a variety of azo dyes significantly depend on the presence of some redox mediators [6,7,43], which is cost consuming. However, using some mediators also leads to the formation of highly unstable radical intermediates that could significantly inactivate laccase [44,45]. In this study, the *P. putida* surface-immobilized laccase system is capable of decolorizing azo dye (AR18) without any redox mediator. This result strongly contrasts with data obtained with some fungal laccases that require mediators for decolorization activity [46-49]. In addition, laccases use the distinctive redox ability of copper ions to catalyze the oxidation of aromatic substrates, thus, whether the system requires additional supplement of copper ions during the reaction was investigated. The results showed that copper ion addition is not necessary for separate or mixed dyes. Therefore, this system is advantageous because of its independence from mediators and copper ions.

High thermostability of an enzyme system is generally considered as an advantageous for industrial enzyme-degrading processes in terms of increasing reaction rate and decreasing mass transfer limitation under high temperatures. Unlike several previously described fungal laccases that had very high optimum reaction temperatures at 50°C to 80°C [50-52], the bacterial laccase WlacD was found thermally stable at 0°C to 25°C, with maximum reactivity at 25°C, but inactivated rapidly above 40°C [31]. The cell platform conferred to the surface-displayed laccase improved thermostability, with respect to the optimum temperature of 35°C and an operational temperature range of 15°C to 45°C shown in this study (Figure 6b). Although the current system is still demarcated as moderate temperature-dependent, an efficient decolorization of either separate or mixed dyes was achieved, given the remarkably high functionality and insignificant mass transfer limitation of the system compared with those that require high temperature to maximize the activity and eliminate mass transfer problem. Therefore, a high degrading efficacy with relatively wide applicable temperatures and without significant mass transfer limitation would be beneficial to validate this engineered system for multipurpose requirements of industrial decolorization or detoxification treatments.

In continuous decolorization experiments, the system exhibited remarkable efficacy and good performance to mixed dyes. A greater increase in decolorization level, selectivity, performance, and regenerability of engineered *P. putida* cells is more likely achieved using a bioreactor or fed-batch process. However, displaying additional thermally stable laccases, such as fungal laccases, onto the surface of target bacteria should also be considered. The development of capacity-promoted *P. putida* surface-displayed laccase systems is now one of our primary goals.

## **Conclusions**

The feasibility of engineered *P. putida* cells with surface-immobilized bacterial laccase for the decolorization of two industrial synthetic dyes has been demonstrated. Different tandem-aligned anchor repeats were used to obtain an optimized cell surface display system. The displayed laccase exhibited high reactivity to either single or mixed dyes, which was performed at 35°C with maximum activity, and were stably conducted across a temperature range of 15°C to 45°C. The decolorization reactions were Cu^2+^- and mediator-independent, but an obligate pH value was required for maximal decolorization. Continuous three-round shake-flask experiments showed that the system retained decolorization efficiency during the first two rounds, and that both decolorization and whole-cell laccase activity can be reverted to initial levels using a simple regeneration process. This study is the first to test a regenerative engineered bacterial system in the biocatalysis of synthetic dyes.

## **Methods**

### **Dyes and chemicals**

**Figure 9:**
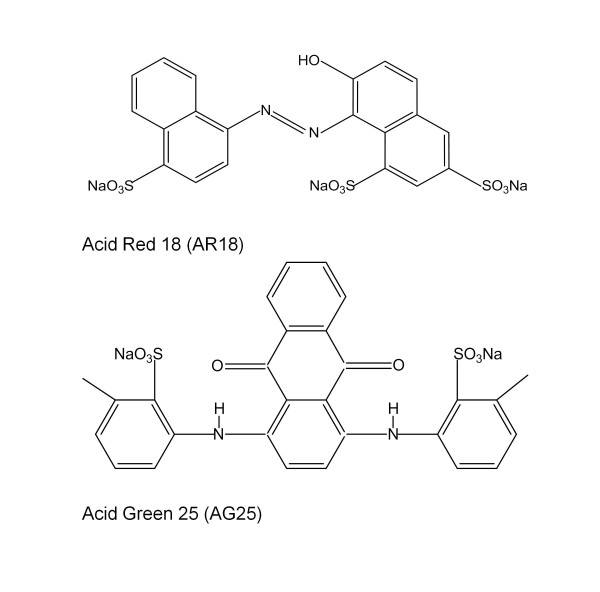
Chemical structures of laccase substrate compounds.

Two industrial grade synthetic acid dyes currently used in textile dyeing, Acid Red (AR) 18 (C_20_H_11_N_2_Na_3_O_10_S_3_) and Acid Green (AG) 25 (C_28_H_20_N_2_Na_2_O_8_S_2_) (Figure 9), were purchased from the Modern Dyestuffs & Pigments Co., Ltd. (Thailand) and used for the dye decolorization analysis without further purification. 2,2’-Azino-bis(3-ethylbenzthiazoline-6-sulfonic acid (ABTS) was purchased from AMRESCO Inc. (USA) and used as substrate for laccase enzymatic activity measurement. Peptone, yeast extract, and other bacteria culture medium ingredients were purchased from Shuangxuan Microbe Culture Medium Products Factory (Beijing, China). All other chemicals were of analytical grade.

### **Bacterial strains, plasmids, and culture conditions**

The bacterial strains and plasmids used in this study are listed in Table 1. Briefly, *E. coli* DH5α (TaKaRa Bio Inc.) cells were used to construct various recombinant plasmids. The wild-type *P. putida* Migula AB92019 was used as host strain for surface display experiments and as negative control strain for relevant analyses. Recombinant plasmids pMB281, pMB282, and pMB283, respectively harboring the fusion gene with *inaQ-N* (encodes the first 175 aa of the InaQ) [35] at 1 to 3 tandem aligned repeats and *wlacD* (encodes a bacterial laccase) [31]*inaQ-N*/*wlacD*, (*inaQ-N*)_2_/*wlacD*, and (*inaQ-N*)_3_/*wlacD*, were constructed for the expression and display of the corresponding fusion proteins.

**Table 1:** Bacterial strains and plasmids used in the current study

**Strains/plasmids**	**Genotype/description**	**Source**
*E. coli* DH5α	*supE*44Δ*lac*U169(Φ80 *lacZ*ΔM15) *hdsR*17 *recA*1 *endA*1 *gyrA*96 *thi-*1 *relA*1	Laboratory stock
*P. putida*		
CCTCC AB92019	Cb^s^, wild-type strain with pronounced vitality in wild environments	CCTCC^a^ stock
MB284	*P. putida* CCTCC AB92019 construct harboring pMB281	This study
MB285	*P. putida* CCTCC AB92019 construct harboring pMB282	This study
MB286	*P. putida* CCTCC AB92019 construct harboring pMB283	This study
Plasmids		
pMB104	Amp^r^Cb^r^, *E. coli–P. putida* shuttle vector containing *P*_*oprL*_ promoter and *inaQ-N*/*gfp* fusion gene, 6142 bp	[35]
pMB111	Amp^r^Cb^r^, *E. coli–P. putida* shuttle vector containing *P*_*oprL*_ promoter and (*inaQ-N*)_2_/*gfp* fusion gene, 6670 bp	Laboratory stock (unpublished)
pMB112	Amp^r^Cb^r^, *E. coli–P. putida* shuttle vector containing *P*_*oprL*_ promoter and (*inaQ-N*)_3_/*gfp* fusion gene, 7198 bp	Laboratory stock (unpublished)
pMB172	Amp^r^; the recombinant plasmid carrying the mutated *wlacD* gene; 8,867 bp	[31]
pMB281	Amp^r^Cb^r^, *E. coli–P. putida* shuttle vector containing *P*_*oprL*_ promoter and *inaQ-N*/*wlacD* fusion gene, 6872 bp	This study
pMB282	Amp^r^Cb^r^, *E. coli–P. putida* shuttle vector containing *P*_*oprL*_ promoter and (*inaQ-N*)_2_/*wlacD* fusion gene, 7404 bp	This study
pMB283	Amp^r^Cb^r^, *E. coli–P. putida* shuttle vector containing *P*_*oprL*_ promoter and (*inaQ-N*)_3_/*wlacD* fusion gene, 7926 bp	This study

^a^ China Center for Type Culture Collection.

All strains were grown in Luria–Bertani medium (LB), unless specified otherwise. Recombinant *E. coli* cells were cultured in LB containing 100 g/ml ampicillin (Amp) at 37°C, whereas recombinant *P. putida* strains were grown in LB containing 500 g/ml carbenicillin (Cb) at 28°C.

### **Plasmid construction and transformation**

Total bacterial DNA was extracted using a standard procedure [53]. The *wlacD* gene was amplified using polymerase chain reaction (PCR) from the plasmid pMB172 [31] with primers F_lac_: 5′−CCGAGATCTATGCAACGTCGTGATTTC−3′ (*Bgl*II site underlined) and R_lac_: 5′−AAAGAATTCTTATACCGTAAACCCTAAC−3′ (*Eco*RI site underlined). The PCR-amplified fragment was sequenced before digestion with *Bgl*II and *Eco*RI. The digested fragment was then ligated to the *Bgl*II/*Eco*RI site of a previously constructed plasmid pMB104 that harbors recombinant *inaQ-N*/*gfp* fusion gene under the control of a constitutive promoter *P*_*orpL*_ (the *gfp* was positioned at the *Bgl*II/*Eco*RI site) [35], and was ligated to the plasmids pMB111 and pMB112 that harbor (*inaQ-N*)_2_/*gfp* and (*inaQ-N*)_3_/*gfp* (data are unpublished) genes, respectively, yielded the plasmids pMB281, pMB282, and pMB283, which harbor the fusion genes *inaQ-N*/*walcD*, (*inaQ-N*)_2_/*walcD*, and (*inaQ-N*)_3_/*walcD*, respectively (Figure 2).

Transformation of *E. coli* was performed following ″Protocol 25″, as described previously [54], whereas the transformation of recombinant plasmids into *P. putida* AB92019 was performed using a previously described method [55].

### **Cell fractionation**

Cell suspensions were passaged twice through a French Pressure Cell (Thermo, USA) at 20,000 psi. The disrupted mixtures were then fractionated following the procedures described previously [36].

### **SDS-PAGE and western blot analysis**

Fusion proteins InaQ-N/WlacD, (InaQ-N)_2_/WlacD and (InaQ-N)_3_/WlacD prepared from whole cell fraction (WC), cytoplasmic fraction (CP), and outer membrane fraction (OM) of the transformed *P. putida* cells were analyzed through SDS-PAGE using 12.5%, 10%, and 10% polyacrylamide gels, respectively. The proteins in the gels were then transferred onto Hybond-polyvinylidene fluoride membranes (Amershan, USA). Western blot analysis was further performed using polyclonal WlacD antiserum [22] as primary antibodies. Other following procedures were as described previously [22].

### **Immunofluorescence microscopy and fluorescence-activated cell sorting (FACS) analysis**

Immunofluorescence microscopic observation and FACS analysis of recombinant *P. putida* cells were performed following previously described procedures [35], except for using polyclonal anti-WlacD antiserum as primary antibodies. FACS measurements were recorded as the percentage of total WlacD-labeled cells relative to the total Cy5 fluorescence.

### **Measurement of whole-cell laccase activity**

Whole-cell laccase enzymatic activity was measured with ABTS as substrate at 25°C following a previously described method [31]. The reaction mixture contained 0.5 mM ABTS, 0.1 M sodium acetate buffer (pH 3.0), 0.1 M CuCl_2_, and a suitable amount of recombinant *P. putida* cells. The whole-cell WlacD enzymatic activity was expressed in units. One unit of enzymatic activity was defined as the amount that oxidized 1 μmol of ABTS per min.

### **Decolorization of separate or mixed dyes**

The absorbance value of dyes was recorded using a UV/VIS spectrophotometer (DU-800 Nucleic Acids/Protein Analyzer, Beckman Coulter). The decolorization of AR18 and AG25 using recombinant *P. putida* cells was tested with and without Cu^2+^ addition. The reaction mixture (5 ml) contained 1 g/l dye (unless stated otherwise), 70 mM sodium acetate buffer (pH 3.0), and the harvested recombinant *P. putida* MB285 cells at a concentration of approximately 1 × 10^8^ cells/ml to 1 × 10^9^ cells/ml. The mixtures were incubated at 25°C and shaken at 200 rpm. The absorbance of the supernates was then spectrophotometrically measured at 506 nm for AR18 and at 639 nm for AG25 at different time intervals. Control samples of *P. putida* AB92019 cells were run in parallel.

For the decolorization experiments of independent AR18 or AG25 at different temperatures (15°C to 85°C) and pH values (pH 3.0, 5.0, 7.0, and 8.5), prior to decolorization determination, the suspensions of *P. putida* MB285 cells were maintained in a water bath until the given temperature is reached or the pH was adjusted into the given value. The reaction was immediately run by adding the cell suspension into a similarly preheated or pH-preadjusted reaction solution. The absorbance of the supernates was then monitored in time course.

For continuous three-round decolorization experiments, the decolorizing activity of the mixed AG25 and AR18 (each 1 g/l at the final concentration of the reaction mixtures) was tested in shake-flask trials at 200 ml reaction solution at pH 3.0, 25°C, 200 rpm shaking, and without Cu^2+^. After the first-round reaction, the cells were harvested via centrifugation and were directly used for the second-round reaction upon similar conditions. The supernate was removed through centrifugation after the second-round reaction, and the 100 ml LB medium was directly added into the flask to allow the growth of residual cells under 25°C, 200 rpm for 4 h without strictly aseptic operations. A third-round decolorization reaction followed under similar reaction conditions after removal of the medium via centrifugation.

The activity was expressed as the relative decolorization value, which was calculated as follows:

Relative decolorization value (%) = [(A_0_ − A)/A_0_] × 100

A_0_ − Initial absorbance

A − Final absorbance.

### **Statistical analysis**

Statistical analysis was performed using the SPSS 13.0 statistical software. All data presented are the averages of at least three assays. Statistical significance was defined as *P* < 0.05.

## **Competing interests**

The authors declare that they have no competing interests.

## **Authors' contributions**

WW and ZZ performed most of the experiments, made most of the data evaluation and interpretation. HN, XY, and QL participated in partial experiments. LL conceived and directed the study as well as prepared the manuscript. All authors read and approved the final manuscript.
